# Differentiation in Emotional Investments in Work Groups among Different Social Status of Construction Industry Practitioners: A Perspective from the Social Exchange Theory

**DOI:** 10.1155/2022/9306167

**Published:** 2022-05-14

**Authors:** Wenqing Zhang, Dingzhou Fei

**Affiliations:** Department of Psychology, Wuhan University, Wuhan, China

## Abstract

The construction industry is characterized by a high level of mobility and a diverse range of practitioners from different social status, which can affect the industry's group management processes. The exploration of the mechanisms involved is an important task for theoretical research and a challenge for management practices. This study examines three relevant aspects of work-group behavior in the construction industry from a social exchange perspective: the individual's evaluation of the level of the emotional investment of members in the work team and their assessment of personal rewards and costs. The study of 71 construction industry workers through the development of a cost-benefit inventory questionnaire of individual-team exchange relationships revealed that their level of emotional investment in the work group can be predicted by assessing their awareness of personal rewards and costs. A further clustering algorithm revealed that an individual's social status had a significant impact on their level of affective investment, but there was no significant correlation between an individual's wage and their level of emotional investment in the work team. The findings deepen our understanding of group behaviors in the construction field by explaining the interactions between individuals and organizations in work groups while emphasizing the indispensable role of emotional factors in group development.

## 1. Introduction

### 1.1. The Current Situation of Construction Industry Workers

After a long period of market system construction and effective regulation and rectification, the construction industry is steadily developing. At the same time, some social problems caused by the nature of the long project cycle, many projects, and complex environment have been exposed. Among them, the emotional problems of workers are coming to the fore more and more. Workers who are highly mobile due to site changes and need to move around all year round are usually far away from the city and their families and friends, and the resulting work-family conflicts can affect the social behavior of construction workers [1], and kinship plays an important role in the effectiveness of relational governance mechanisms in projects [2]. In addition, construction industry workers have a complex composition and diverse social backgrounds and are forced to accomplish demanding and urgent tasks in an undesirable living environment and monotonous life. In such a work and life environment, it is imperative to pay attention to the emotional state of construction workers who are motivated by different social classes [3]. In addition, the current external competition of construction enterprises is becoming increasingly fierce, and in order to cope with the external pressure, enterprises need to give full play to their talents and mobilize the emotional engagement of their employees to cope with the market demands.

### 1.2. Social Exchange Theory

As one of the major theories of social interaction in the social sciences, theoretical and empirical applications involve extending the work of social exchange theory to the analysis of power and dependency, social networks, reciprocity, equity, social cohesion, and solidarity [4].

Airman and Taylor [5] studied relationship development from the perspective of social penetration, which led to the formulation of the well-known social exchange model of interpersonal interactions. Social penetration is viewed as a systematic process of self-revelation among individuals in interpersonal interactions in which people continually assess the quality of their interactions to determine whether they need to continue to invest. In contrast, the behaviors exhibited in social systems are the result of individual decision-making, where group members exchange various types of resources based on their assessment of and dependence on others. In previous research, the social exchange and exchange of information among construction industry practitioners can influence decisions on construction projects, which in turn affect work health and safety [6]. In addition, components of the emotional exchange model are often used to explain dynamic relationships such as emotional experience and expression [7]. It was also found that interpersonal relationships are continuously socially exchanged in a continuous developmental process and have a tendency to be increasingly rewarding.

### 1.3. Emotional Investment

Emotional investment is a relational orientation in which individuals show loyalty and commitment to their work group and show mutual concern for their colleagues, and trusting interactions between participants help foster beneficial relational behaviors that in turn improve team performance [8]. It helps to improve team communication and thus effectively increases team effectiveness [9] As members' mutual interest increases, personal relationships accumulate and relationships with others become closer, and individuals make ongoing emotional investments in relationships [10], while each successful social exchange increases interdependence and commitment among work group members [11]. With increased interdependent communication, more and more team members have a higher level of emotional investment, which in turn leads to more effort for the team's goals and increases the satisfaction of other members. At the same time, the team environment evolves away from the previous mutual exchange of resources among group members to one in which the individual needs of members are met within the work group. Current research hot spots for emotional investment are applications to family groups of foster children [12, 13] and social media [14, 15], but we believe emotional investment can be applied to a broader range of domains.

### 1.4. Personal Rewards and Personal Costs

Personal rewards refer to enjoyable or appreciated relational attributes that an individual receives in a relationship. Rewards are usually divided into six categories: money, status, love, information, things, and services. Each person defines rewards differently; what one person seeks may be worthless to another. Personal costs refer to the relationship attributes that individuals pay for in relationships that are annoying or disliked. For social exchange theory, successful resource exchange means that rewards can be maximized in exchange for the smallest possible personal cost. When this ideal social exchange is achieved, group members increase their mutual contribution and commitment (e.g., [16]). Reciprocity signifies engagement and attachment among members, and the results of Xerri's [17] self-reported survey of 255 Australian engineering asset management employees indicated that the organizational support for members can positively predict employees' emotional attachment to the organization. Moreover, organizational support, emotional commitment to the organization, and employees' psychological well-being are mutually reinforcing, so individual rewards may be a more reliable predictor of reciprocity than costs. Therefore, we consider that personal rewards may be a better predictor of emotional investment than personal costs.

### 1.5. Adopting a Social Exchange Perspective to Analyze the Construction Industry

To analyze in depth the attachment and especially the loyalty of construction workers to their groups, we applied social exchange theory to workers and managers in the construction industry, and to examine what characteristics this affective investment has due to the high mobility of the construction industry and how it relates to the social status of construction workers, an important aspect of construction project management theory has to be questioned. Our study is dedicated to find the correlation between social status and emotional investment in the context of a highly mobile profession like the construction industry. Behavior can be seen as the result of an individual's decision to make a trade-off between rewards and costs, a strategic framework for a game in which individuals or groups interact with society and the environment.

We emphasize the role of emotional investment in promoting work group survival and interdependence among team members, as quality relationships within the work group are the foundation for unlocking the individual potential for improved performance in the project environment [18]. In contrast, most studies on work groups emphasize group efficiency and output without examining the costs and rewards perceived by individual group members or the emotional investment members make in the group [19]. But an overemphasis on group output in the short term can have negative effects on group members' psychological well-being and performance and even on the long-term development of the group [20]. The reasons for this are as follows: first, if individuals feel a lot of pressure to perform in the short term and do not receive the emotional attention or personal rewards that are commensurate with the cost, group continuity is difficult to maintain and the long-term development of the group is not guaranteed [21], and group members may choose to leave, so long-term work group performance will be negatively affected. Second, if short-term team performance goals are overemphasized and members are required to sacrifice their personal interests to achieve them, members may reduce their efforts and knowledge contributions to the team [22], and in this case, although members do not terminate their ties to the group, their social interactions are reduced, individual emotional ties with other members are neglected, and the performance of the work group is similarly affected. Emotional support such as motivation has been found to help with multiproject management in the industry, reduce burnout, and help retain project members [23].

As a human resource that needs to be developed in the current situation, the emotions of employees can have a significant impact on the dedication of corporate workers and the development and implementation of corporate systems [24]. In a study of the stress and burnout tendencies of members of different project groups, Pinto [25] found that project managers and construction workers had significantly higher levels of emotional exhaustion relative to other job types, so the construction industry should focus more on the social stratification of the practitioners in order to provide emotional de-escalation and management for groups vulnerable to psychological problems [3]. In view of the special characteristics of the work of employees in the construction industry, part of the employees are migrant worker groups with high job mobility and relatively high wage levels for some skilled workers with little room for promotion, and part of the employees are regular employee groups in construction enterprises with more stable jobs and relatively low wages, but with room for promotion within the enterprises. However, due to the inevitable existence of social prejudice, this paper classifies the group of migrant workers or contract workers as a group with relatively low social status and the group of regular employees within the enterprise as a group with relatively high social status. This paper examines team members' evaluations of personal costs and rewards and their own emotional investment in the work group and compares whether there are significant differences in the levels of emotional investment between the two groups and how their evaluations of personal costs and rewards affect their levels of emotional investment. In a social group, people with high social status usually interact emotionally with the group more than people with low status in the group.

In summary, this study focuses on the role of emotional investment generated by construction industry practitioners in the work group and its influencing factors. We consider the emphasis on affective investment and the focus on stratification of construction workers and the work environment as the main contributions of this study.

Based on the above statements, we build on the construction industry group and adopt a social exchange perspective to propose three hypotheses.

(H1) People with high socioeconomic status have a higher level of emotional investment in the group
(H2) High income does not affect the level of the emotional investment of individuals in the work group
(H3) Personal rewards can be a better predictor of emotional investment than personal costs

## 2. Methods

The methods of this work consist of participants for surveys, questionnaires revised for Chinese contextures, and clustering analysis for defining the major factor affecting the emotional investments in the work groups.

Participants: due to the low participation of women in the architectural, engineering, and construction industry [26], a total of 71 (including 12 women) construction workers were selected to participate in this questionnaire for this study.

Objective of the surveys: to collect construction workers' level of emotional investment in work teams and team members' assessments of individual costs and personal rewards (including regular, contract, and migrant workers) by questionnaire survey method.

Study steps: this task focused on developing questionnaires and scales. The original scales and questionnaires were in English, and to translate all survey items from English to Chinese, we used the translation/back-translation procedure of Brislin [27] and then adapted them to the Chinese context. Finally, the adapted questionnaires and scales were tested for reliability and validity.

To test our proposed hypotheses, we conducted analyses at the individual level. We referred to Saavedra and Dyne [28] and selected three separate items from the cost-benefit inventory developed by [29, 30] based on social exchange in interpersonal relationships to assess individuals' perceptions of rewards and costs. Considering the purpose of this study is to examine the personal trait attributes and relational attributes of the different social status of practitioners within the construction industry, the items focus on the exchange of nonmaterial resources [28]. To measure affective investment, we used three items from Hackman's [21] assessment of members' perceptions of group well-being. A principal components analysis was conducted on nine items to ensure that the scale we used would achieve the predicted effect. The results indicated that the three factors, emotional investment, rewards, and costs, together accounted for 77% of the variance.  Table 1 examines the suitability of the scales we used for principal components analysis, and Table 2 lists the individual items and the factor loadings for each item.

This study used the KMO (Kaiser–Meyer–Olkin) test to statistically compare the correlation coefficients between the variables, which yielded a KMO value of 0.758, which is greater than 0.7 (KMO takes values between 0 and 1, and the larger its value, the more suitable the original variables are for factor analysis). In addition, Bartlett's test *P* < 0.05, the above data indicate that it is appropriate to do factor analysis on the above items.

In addition, we conducted separate reliability analyses for the items measuring each factor in the scale, and Cronbach's *α* coefficients for each item measuring the three factors of emotional investment, personal cost, and personal reward were 0.857, 0.739, and 0.893, respectively, all greater than 0.7, meaning that the reliability of each item in the questionnaire was high.

## 3. Results

To test whether social status affects the level of individual affective investment, we conducted an independent sample *t*-test on the overall affective investment level between the regular and migrant worker groups, and the results are reported in Tables 3 and 4.

From the results of the independent sample *t*-test, it can be concluded that within the confidence interval of 99%, the level of emotional investment between regular and migrant worker groups is significantly different, and the level of the emotional investment of regular workers is significantly higher than the level of the emotional investment of migrant workers in work groups (The Emotional Investment Questionnaire items were selected on a seven-point scale, with a choice of 1 meaning strongly agree and a choice of 7 meaning strongly disagree). The formal worker group and the migrant worker group have different socioeconomic status, and it follows that socioeconomic status affects the emotional investment of individuals in the work group, and individuals with higher socioeconomic status have higher emotional investment in the work group; therefore, H1 is confirmed.

**Figure 1:**
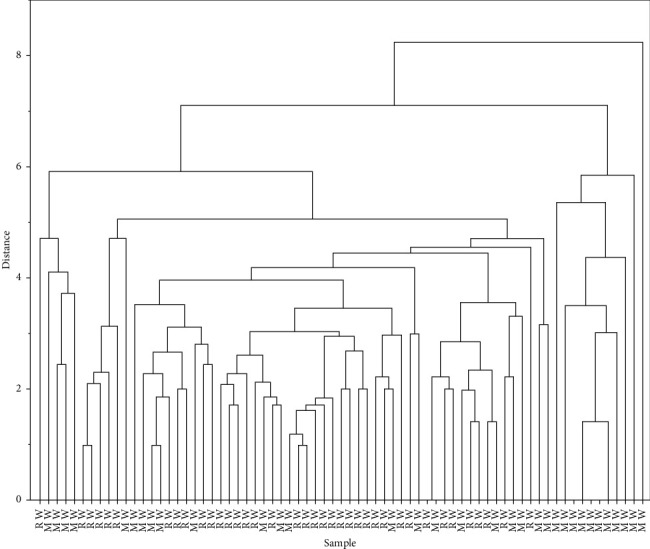
Intergroup differentiation in the level of emotional investment between regular workers and migrant workers through cluster analysis (RW = regular workers, MW = migrant workers).

In order to describe the intergroup differences more intuitively in the overall level of emotional investment in different social status within the construction industry, we conducted a cluster analysis of the results of the emotional investment survey, the results of our survey on sentiment investment were analyzed in Origin using the K-mean algorithm for clustering. As a mainstream data analysis software, Origin has two main functions: data analysis and plotting. Origin's data analysis includes a variety of sophisticated mathematical analyses such as statistics, signal processing, image processing, peak analysis, and curve fitting. We use K-means, an unsupervised learning algorithm for solving clustering problems. The K-means method is a classical algorithm in clustering, one of the top ten classical algorithms for data mining; the algorithm receives the parameter *k* and then divides the *n* data objects entered in advance into *k* clusters, in order to satisfy that the objects in the clusters are more similar, while the objects in different clusters are less similar. The idea of the algorithm is to cluster the *k* points in the sample space, grouping the objects closest to them and updating each cluster centre one by one by iterative methods. The results are shown in Figure 1.

From the results of the cluster analysis, the distance of the vertical coordinate represents the difference in the level of emotional investment between the samples, and the horizontal coordinate represents the individuals; it can be seen that individuals belonging to the same social class are more aggregated and show more similarity in emotional investment, thus concluding that the social stratification in the construction field is obvious, and different groups based on different social classes differ in the characteristic of emotional investment level, while within the group, this characteristic is similar. The characteristics are similar within groups.

In addition, this study tested whether the level of personal income affects the emotional investment of individuals in the work group. The mean of the income levels of the 71 construction industry workers collected in this study was 1068.5 US$, so we used 1068.5 US$ as a cut-off for high- and low-income levels, with those below the mean income considered as a low-income group and those above the mean income considered as a high-income group, and conducted independent sample *t*-tests on the overall emotional investment levels of the two groups. The results are reported in Tables 5 and 6.

As can be seen in Table 6, the difference in the overall level of emotional investment between the high-income group and the low-income group is not significant at a confidence interval of 99%, indicating that there is no significant effect of high income on the level of the emotional investment of individuals; therefore, H2 is proved.

We used hierarchical regression analysis to test H3, and the results are shown in Table 7. When both costs and rewards were regressed on affective investment, only rewards contributed unique and significant variance, indicating that individual rewards were the best predictor of affective investment among work groups in the construction industry; therefore, H3 is confirmed.

## 4. Discussion

Our goal is to illustrate the dynamics of relationships in the construction industry's work community on an emotional dimension, integrating concepts such as satisfaction, investment, and commitment. In the construction industry, construction workers are usually part of a work team, and poor work team relationships predict the work stress of construction workers, and their psychological perceptions greatly influence their behavioral and safety performance, and it is particularly important to study the impact of the work team on construction workers as key and indispensable contributors to each construction project [31]. Research sponsored by the American Society for Quality Control in conjunction with Fortune 500 companies shows that employees see teams as both a platform for personal development and a way to promote [32]. Studies have shown that construction workers with different levels of perception of group norms exhibit different personal safety behaviors at work. In addition, when construction workers were in different groups (e.g., work groups, projects), their perceptions of identity showed significant differences, thus introducing the idea that social identity with a group can moderate the effect of group norms on construction workers' personal safety behavior standards [33]. These findings also suggest new ideas for the management of the construction industry: improving the level of social identification and emotional investment of construction workers in their work group, which in turn improves communication in the team, and effective communication is an important factor in improving team effectiveness, thus enabling construction workers to have better emotional states while improving construction worker safety behaviors and increasing the efficiency of construction project completion.

Furthermore, most research on social exchange has focused on the cognitive aspects of relationships and has mostly examined binary exchanges between individuals, rarely addressing the affective aspects of social exchange. Therefore, this study examines the affective aspects of social exchange in structurally diverse pluralistic work groups within construction companies, extending the scope of the application of social exchange theory. However, excessive emotional attention may lead to a marked decrease in group members' openness to dissenting opinions [34]. By ignoring and excluding different insights and irreconcilable information, groups may make decisions that have serious adverse consequences. These problems are particularly evident in the construction industry, where the completion of projects in the construction industry relies on a variety of task environments such as raw materials, construction personnel, and market research in order to achieve them. Therefore, a balance between efficiency and emotion is needed to solve these problems.

The limitations of this paper are mainly reflected in the following aspects, which we will improve in our future research: firstly, due to the special nature of the sample of construction industry practitioners, the sample size we collected was not very large, but in future research, we will try our best to seek the cooperation of relevant construction companies in the hope that a larger sample size can be obtained. Also, during the data collection process, we discovered an interesting phenomenon: the vast majority of those working in the construction industry are men. This gap persists despite efforts to close the gender gap in the construction and engineering workforce and advocate for gender diversity over the past few decades [35, 36]. Outside of gender, age may have an impact on respondents' level of emotional investment [37], which needs to be refined in the future research. Perhaps in a more in-depth study in the future, we can make a more nuanced segmentation of construction industry practitioners to further explore the social exchanges they engage within their work groups. Finally, this study did not include team performance in the overall structure, and in further research, we plan to explore the impact of emotional investment in work teams on team performance and create a more comprehensive research system considering that emotional investment may cause individuals to overestimate their actual abilities [38].

## Figures and Tables

**Table 1:** KMO and Bartlett test.

KMO measure of sampling adequacy		0.758
Bartlett's test of sphericity	Approx. chi-square	397.199
	*df*	36
	Sig.	0.000

**Table 2:** Factor analyses of self-report items.

Items	F1 personal rewards	F2 emotional investment	F3 personal costs
1. The members of my work team listen to me.	0.831	0.208	0.080
2. I feel needed by my work team.	0.863	0.376	0.068
3. I feel comfortable when I voice my work in work group.	0.835	0.348	079
4. Our team members have a strong sense of loyalty to the team.	0.235	0.925	0.010
5. Our work team members care about the collective and are committed to making it better.	0.370	0.888	0.021
6. I do not want to change the members of our work group.	0.442	0.597	0.128
7. Since I am friends with the members of my work team, I undertake a lot of extra responsibilities.	0.210	0.205	0.832
8. I often compromise my ideas by considering the preferences of other team members.	0.084	0.064	0.826
9. To integrate into the work group, I must sacrifice a great deal of personal independence.	0.223	0.043	0.765

**Table 3:** Analysis of social status among construction workers.

		Overall emotional investment level		
	*N*	Mean	SD	Std. error mean
Regular worker	36	6.8333	3.48727	0.46601
Migrant worker	35	6.8857	3.28194	0.49477

**Table 4:** Differentiation in the level of emotional investment across social classes is shown by *t*-test.

		*t*-test for equality of means						
		*t*	*df*	Sig (2-tailed)	Mean difference	Std. error difference	99% confidence interval of the difference	
							Lower	Upper
Overall emotional investment level	Equal variances assumed	−5.76	69	0.000	−3.80049	0.66535	−5.5926	−2.0677
	Equal variances not assumed	−5.76	44	0.000	−3.80049	0.69693	−5.7071	−1.9532

**Table 5:** Analysis of the income level of construction workers.

			Overall emotional investment level		
	Monthly income level	*N*	Mean	SD	Std. error mean
High-income group	≥1068.5 US	36	6.8333	3.48727	0.46601
Low-income group	<1068.5 US	35	6.8857	3.28194	0.49477

**Table 6:** The relationship between income level and emotional investment is illustrated by *t*-test.

		*t*-test for equality of means						
		*t*	d*f*	Sig (2-tailed)	Mean difference	Std. error difference	99% confidence interval of the difference	
							Lower	Upper
Overall emotional investment level	Equal variances assumed	−0.7	69	0.98	−0.05238	0.80755	−2.1916	2.0868
	Equal variances not assumed	−0.7	69	0.948	−0.05238	0.80709	2.1903	−2.0856.

**Table 7:** Hierarchical regression analysis of emotional investment and personal costs and rewards.

Dv	IV	Adj. *R*^2^	*F*	Beta for IV's
Emotional investment	Rewards	0.472	62.566	0.692
Emotional investment	Costs	−0.008	0.421	0.078

Emotional investment	Rewards	0.464	30.883	0.690
	Costs			0.022

## Data Availability

The [data type] data used to support the results of this study are available from the corresponding author upon request.
